# Occupational Grade, Mental Distress, and the Use of Psychotherapy

**DOI:** 10.1177/21501319231199958

**Published:** 2023-09-20

**Authors:** Lauri Kokkinen, Kia Gluschkoff, Johanna Kausto, Sanna Selinheimo, Kaija Appelqvist-Schmidlechner, Päivikki Koponen, Ari Väänänen

**Affiliations:** 1 Tampere University, Tampere, Finland; 2 Finnish Institute of Occupational Health, Helsinki, Finland; 3 Finnish Institute for Health and Welfare, Helsinki, Finland

**Keywords:** socioeconomic status, occupational grade, mental health, mental distress, psychotherapy, rehabilitation

## Abstract

**Introduction:**

Previous studies have shown that manual workers use less psychotherapy than non-manual workers. However, little is known about the match between the use and the need of psychotherapy in different occupational grades. Our study investigates how the prevalence of mental distress corresponds to psychotherapy use rate in different occupational grades by gender.

**Methods:**

The data were collected from the Rise of Mental Vulnerability Study (use of psychotherapy) and the FinHealth 2017 Study (prevalence of mental distress). Adjusting for age, we calculated General Health Questionnaire (GHQ-12) caseness (a measure for mental distress), a 3-year psychotherapy use rate, and the ratio between GHQ caseness and the psychotherapy use rate in 3 occupational grades (upper non-manual employees, lower non-manual employees, and manual workers) for men and women separately.

**Results:**

In men, for 1 person having used psychotherapy there were 10 persons experiencing mental distress in upper non-manual workers, 14 in lower non-manual workers, and 31 in manual workers. In women, for 1 person having used psychotherapy, there were 6 persons experiencing mental distress in upper non-manual workers, 9 in lower non-manual workers, and 18 in manual workers.

**Conclusions:**

At the population level, manual employees use considerably less long-term psychotherapy than upper non-manual workers although their level of mental distress is high. This indicates a mismatch between symptoms and therapy, which was higher for men in all occupational grades.

## Introduction

Previous studies have shown that manual workers (eg, electricians, plumbers, carers, and cleaners) tend to use less psychotherapy than non-manual workers (eg, teachers, journalists, nurses, and salespersons).^1^ This gap may be linked to individual factors, such as lower mental health literacy—which reduces help-seeking and the willingness to pay for psychotherapy—as well as external factors, such as availability, accessibility, and the cost of psychotherapy.^2^

However, compared to non-manual workers, the need for psychotherapy may be the same or even higher among manual workers.^3,4^ Due to this possible mismatch between the use and need of psychotherapy, we compared the use of psychotherapy and mental distress rate in different occupational grades.

In Finland, rehabilitative psychotherapy is a statutory right granted to all upon referral from a psychiatrist determining someone as being at risk of work disability due to mental disorders. However, psychotherapy is only partly compensated by social insurance—that is, a fixed amount covering roughly half the cost, depending on the fee of the therapist—and obtaining this service takes effort due to a shortage of psychotherapists.^5,6^ Therefore, we predict a relatively large gap in the use of psychotherapy between different occupational grades in Finland.

## Materials and Methods

### Data

Data for rehabilitative psychotherapy use were collected from the Rise of Mental Vulnerability Study, in which a 33% random sample of the working-age population (18–64 years) was drawn from the Statistics Finland population database from 2016 (N = 632 192). Information on reimbursed psychotherapy that began between 2017 and 2019 was obtained from the Social Insurance Institution. The socio-demographic characteristics (age, gender, and occupation) of the sample were obtained from 2016.

Data for mental distress were collected from the FinHealth 2017 Study implemented by the Finnish Institute for Health and Welfare in which a random sample of the adult population (over 18 years of age) was drawn from the population database (N = 10 247).^7^ The data were gathered using self-administered questionnaires, and the response rate was 69%, with 7050 respondents. From these respondents, we selected those who were under 61 years of age in 2016, were working, and had answered the General Health Questionnaire (GHQ), which is a psychometric screening tool for identifying mental distress.^8^ The data on age, gender, and occupation were obtained from the population database.

### Measures

Rehabilitative psychotherapy use was defined as a dichotomous measure (1 = yes and 0 = no). GHQ caseness was used to indicate individuals with mental distress. GHQ caseness (1 = yes and 0 = no) was calculated using 4 points as the threshold for mental distress (in the 12-item GHQ, the outcomes vary between 0 and 12). For occupational grades, we chose upper non-manual employees, lower non-manual employees, and manual workers using a Statistics Finland classification system. Gender was treated as a dichotomous variable and age as a continuous variable.

### Statistical Analysis

Adjusting for age, we calculated GHQ caseness, the (3-year) psychotherapy use rate, and the ratio between GHQ caseness and the psychotherapy use rate in the 3 occupational grades separately for men and women. Age adjustment was performed through regression analysis by using regression model-generated values of GHQ caseness and the psychotherapy use rate at the average sample age of 40 years. Analyses were performed using R version 4.1.2.^9^

## Results

**Figure 1.**
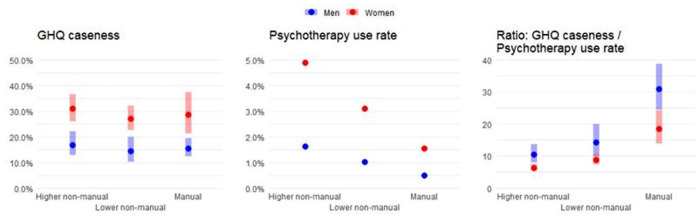
GHQ caseness, the psychotherapy use rate, and the ratio between GHQ caseness and the psychotherapy use rate in 3 occupational grades.

Table 1 shows the socio-demographic characteristics of the 2 study populations (Figure 1).

**Table 1.** The Socio-Demographic Characteristics of the 2 Study Populations.

	FinHealth 2017 study	Rise of mental vulnerability study
N	2145	632 192
Women	51%	52%
Men	49%	48%
Age (mean (SD))	39.98 (11.43)	40.14 (11.69)
Occupational grade		
Higher non-manual	28%	25%
Lower non-manual	40%	41%
Manual	32%	34%

Mental distress (as measured by GHQ caseness) was highest for the higher non-manual grade and lowest for the lower non-manual grade among both women and men. These differences were, however, very small. The psychotherapy use rate showed a clear gradient from the higher non-manual grade to the manual grade for both genders.

In the group of higher non-manual male workers, there were 10 persons who experienced mental distress for 1 person having undergone psychotherapy. For lower non-manual and manual male workers, the numbers were 14 and 31, respectively. In the group of higher non-manual female employees, there were 6 persons with mental distress for 1 person having undergone psychotherapy. For lower non-manual and manual female workers, these numbers were 9 and 18, respectively.

## Discussion

We found a large discrepancy between the use of psychotherapy and the potential need for treatment between occupational grades. At the population level, manual employees use considerably less rehabilitative psychotherapy than upper non-manual workers although their level of mental distress is high, indicating a mismatch between symptoms and treatment. Revealing this mismatch between the use and need of psychotherapy is important, as psychotherapy uses scarce health care resources in an intensive way and is partially tax-funded in Finland.

In this study, mental distress did not follow a clear occupational gradient. This may be because mental health is related to several socio-cultural characteristics, such as social class-related habits and gendered occupational conditions.^10,11^ Instead, the mismatch between the use and need of psychotherapy was due to the lower use of psychotherapy among manual and lower non-manual workers.

Both mental distress and the use of psychotherapy were higher among females in all occupational grades, as also suggested by previous studies.^2^ The new finding was that there was a considerable mismatch between the use of psychotherapy and the level of mental distress for men throughout the occupational grades. Due to the limited sample size regarding the data on mental distress, however, we suggest that these findings should be confirmed with larger datasets.

Previous studies have shown the efficacy of psychotherapy for different mental health problems.^12-14^ According to our study, higher occupational grades have been more able to implement this scientific evidence to relieve their mental distress. Even though Finland has universal health coverage, psychotherapy clients pay roughly half of the service out of pocket,^5^ and we suspect this reliance on personal finance accentuates the occupational gradient in the mismatch between the use and a potential need of psychotherapy. Furthermore, it is possible that psychotherapy for some individuals in higher occupational grades would be covered by private insurance through their employer, while manual workers would not have access to this benefit.

Even if previous studies have shown the efficacy of psychotherapy for different mental health problems in general population,^12-14^ less is known about the gender-related effect of long-term psychotherapy among manual worker men and women. There is some evidence that a combination of various practical, psychological, and cultural barriers influences treatment engagement and gaining from psychotherapy among employees with lower occupational grade.^15^ A recent study on disability trajectories of rehabilitative psychotherapy recipients also found that manual workers who received therapy were more likely to remain on the path of prolonged disability than non-manual employees who received similar therapy.^16^ In future studies we should, however, pay more attention to gender and also explicitly test the possibly cumulative effects of occupational grade and gender on gains from psychotherapy. For doing this, we need to move beyond the logic of conventional net effect analyses which estimate the isolated impact of individual variables on treatment outcomes, and instead focus on the effects of different occupational grade-gender configurations by using for example fuzzy-set analysis.^17^

We conclude that more emphasis should be placed on barriers to mental health treatment among men and lower occupational grades. Our results may indicate that psychotherapy does not fit the needs of all working-age population, and we suggest that they should be developed and offered alternative methods of rehabilitation.
